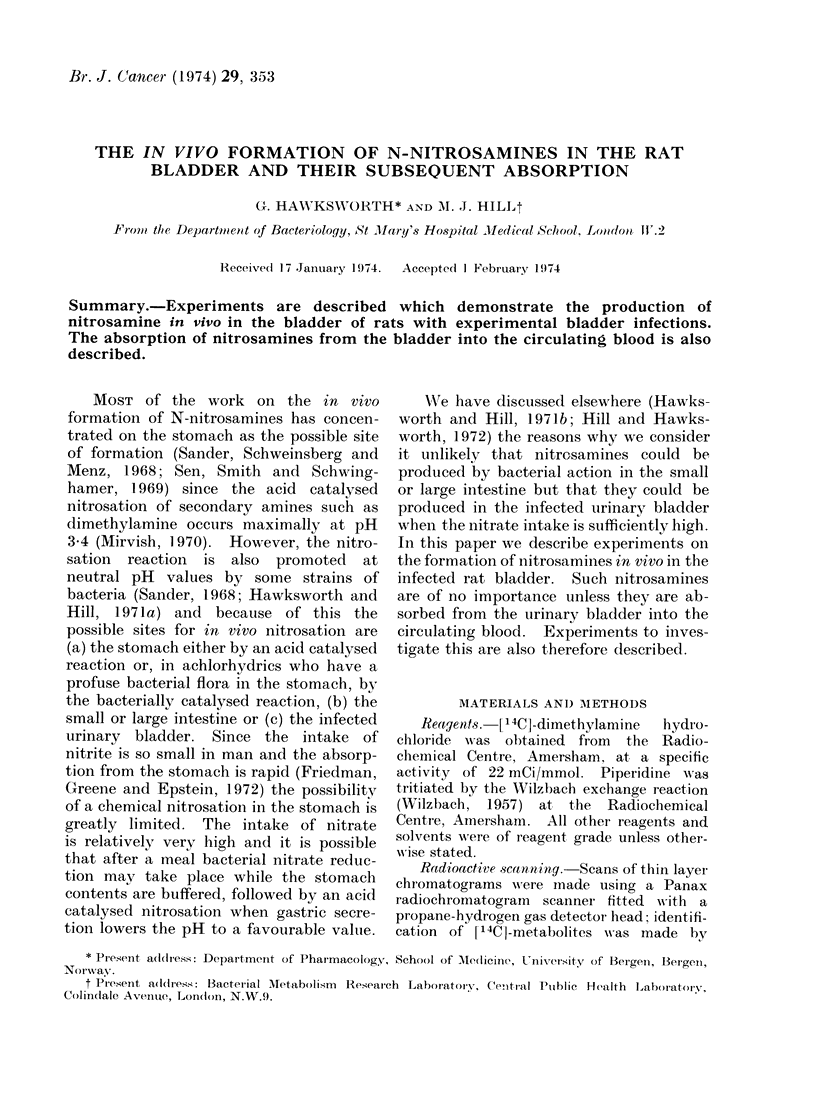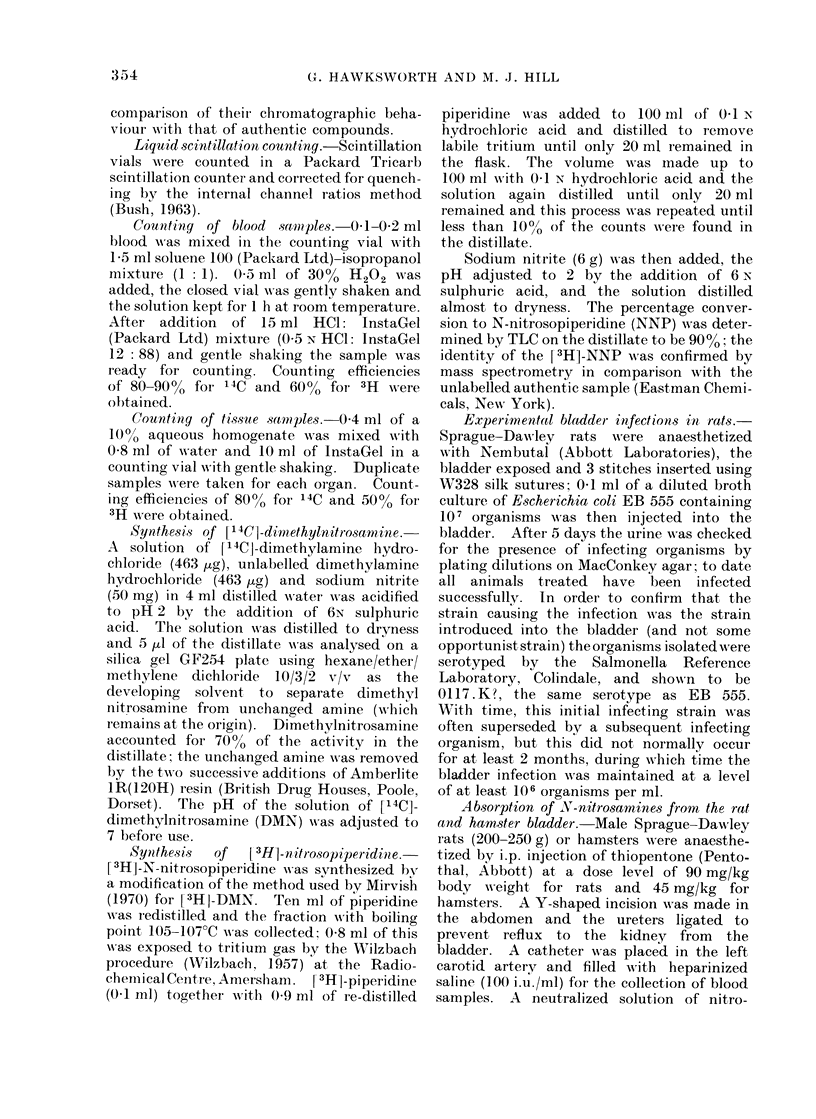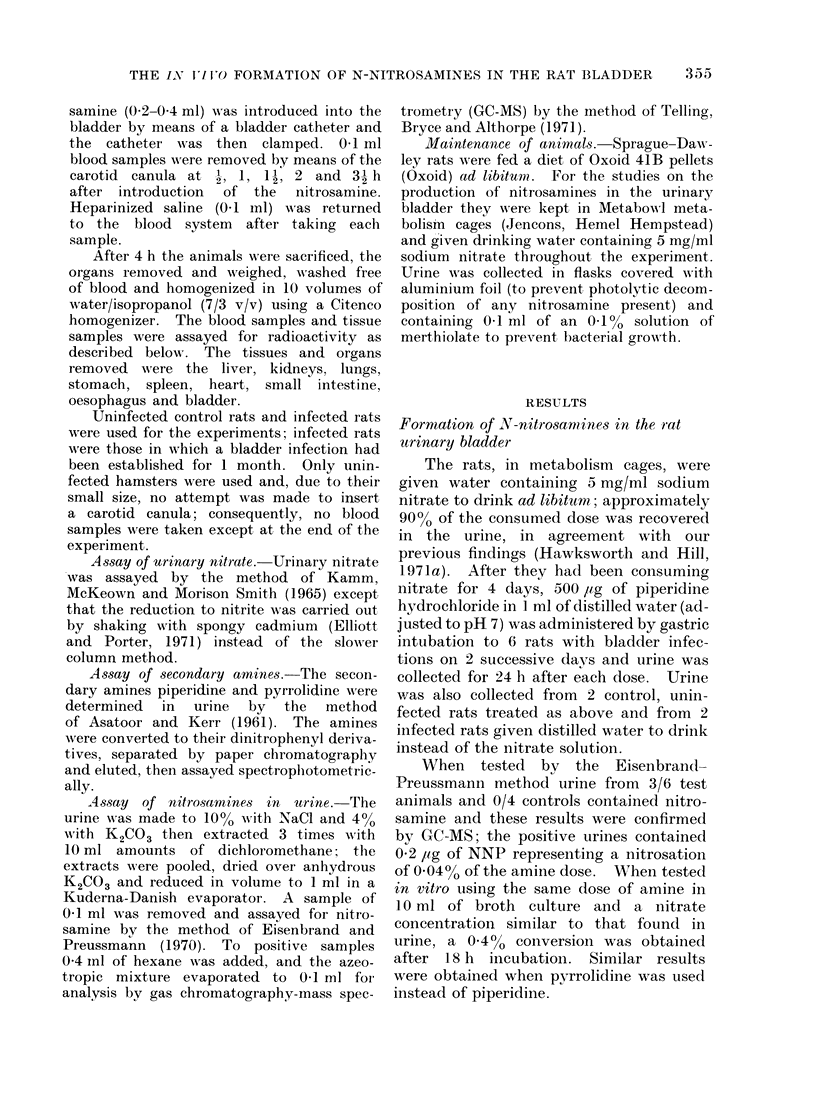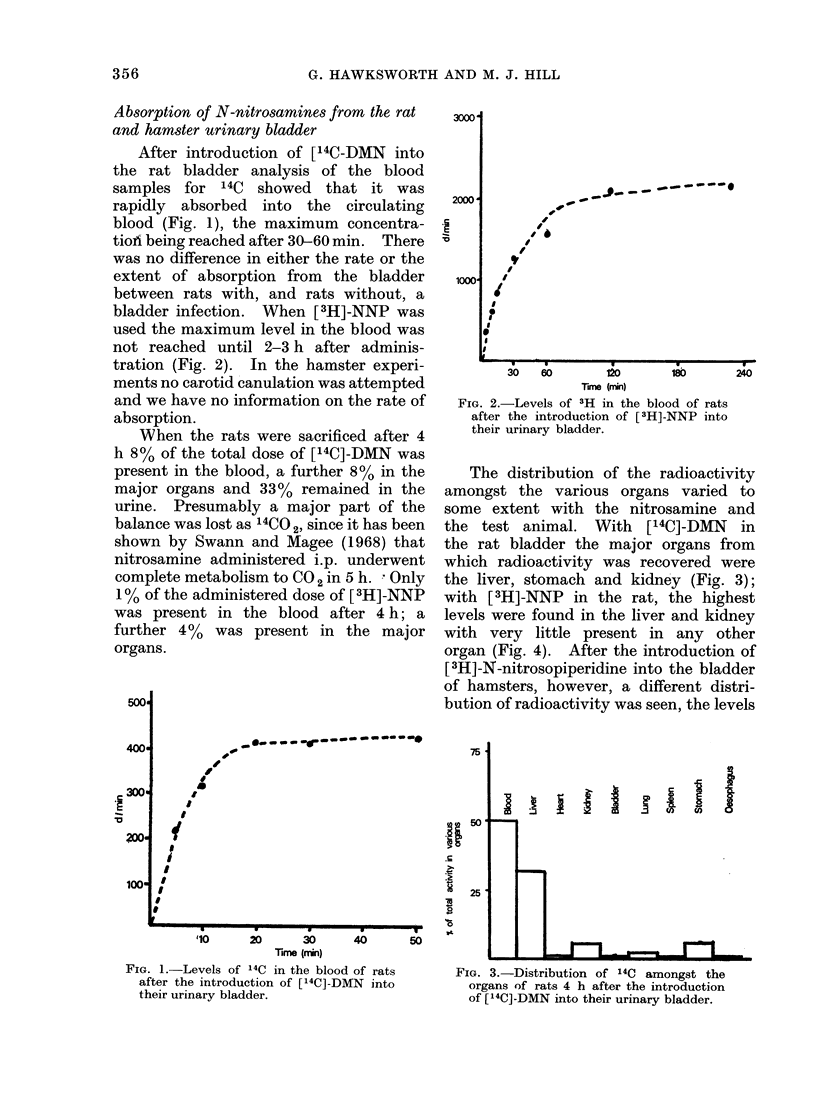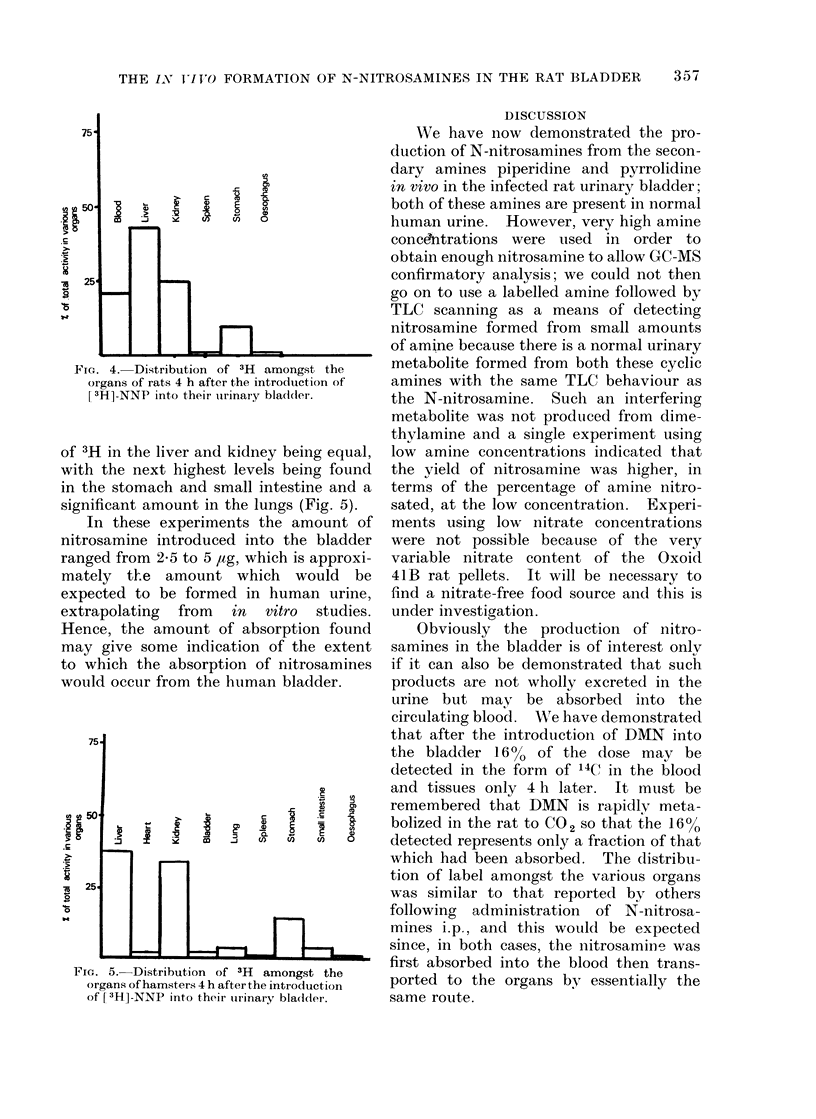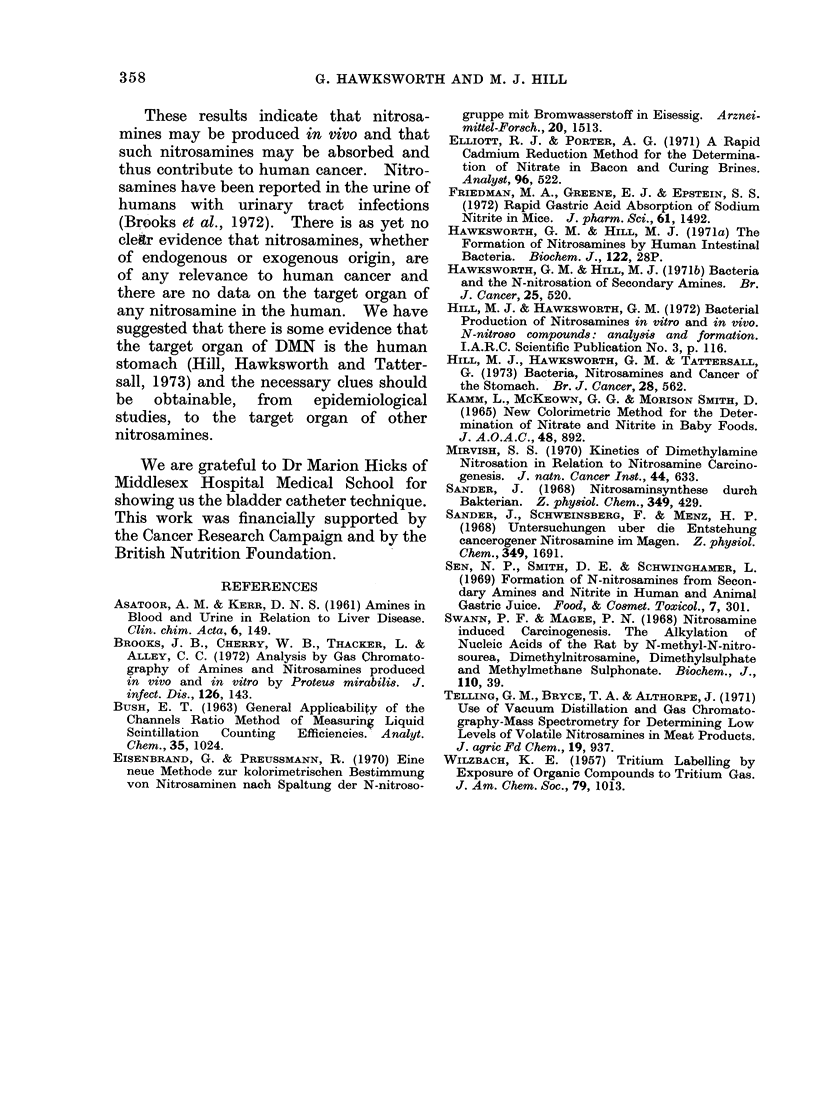# The in vivo Formation of N-Nitrosamines in the Rat Bladder and their Subsequent Absorption

**DOI:** 10.1038/bjc.1974.82

**Published:** 1974-05

**Authors:** G. Hawksworth, M. J. Hill

## Abstract

Experiments are described which demonstrate the production of nitrosamine *in vivo* in the bladder of rats with experimental bladder infections. The absorption of nitrosamines from the bladder into the circulating blood is also described.


*Br. J. Cancer* (1974) **29**, 353

### THE *IN VIVO* FORMATION OF N-NITROSAMINES IN THE RAT BLADDER AND THEIR SUBSEQUENT ABSORPTION

G. HAWKSWORTH* AND M. J. HILL†

*From the Department of Bacteriology, St Mary's Hospital Medical School, London W.2*

Received 17 January 1974. Accepted 1 February 1974

**Summary.—Experiments are described which demonstrate the production of nitrosamine *in vivo* in the bladder of rats with experimental bladder infections. The absorption of nitrosamines from the bladder into the circulating blood is also described.**

MOST of the work on the *in vivo* formation of N-nitrosamines has concentrated on the stomach as the possible site of formation (Sander, Schweinsberg and Menz, 1968; Sen, Smith and Schwinghamer, 1969) since the acid catalysed nitrosation of secondary amines such as dimethylamine occurs maximally at pH 3·4 (Mirvish, 1970). However, the nitrosation reaction is also promoted at neutral pH values by some strains of bacteria (Sander, 1968; Hawksworth and Hill, 1971*a*) and because of this the possible sites for *in vivo* nitrosation are (a) the stomach either by an acid catalysed reaction or, in achlorhydrics who have a profuse bacterial flora in the stomach, by the bacterially catalysed reaction, (b) the small or large intestine or (c) the infected urinary bladder. Since the intake of nitrite is so small in man and the absorption from the stomach is rapid (Friedman, Greene and Epstein, 1972) the possibility of a chemical nitrosation in the stomach is greatly limited. The intake of nitrate is relatively very high and it is possible that after a meal bacterial nitrate reduction may take place while the stomach contents are buffered, followed by an acid catalysed nitrosation when gastric secretion lowers the pH to a favourable value.

We have discussed elsewhere (Hawksworth and Hill, 1971*b*; Hill and Hawksworth, 1972) the reasons why we consider it unlikely that nitrosamines could be produced by bacterial action in the small or large intestine but that they could be produced in the infected urinary bladder when the nitrate intake is sufficiently high. In this paper we describe experiments on the formation of nitrosamines *in vivo* in the infected rat bladder. Such nitrosamines are of no importance unless they are absorbed from the urinary bladder into the circulating blood. Experiments to investigate this are also therefore described.

### MATERIALS AND METHODS

*Reagents.*—[$^{14}$C]-dimethylamine hydrochloride was obtained from the Radiochemical Centre, Amersham, at a specific activity of 22 mCi/mmol. Piperidine was tritiated by the Wilzbach exchange reaction (Wilzbach, 1957) at the Radiochemical Centre, Amersham. All other reagents and solvents were of reagent grade unless otherwise stated.

*Radioactive scanning.*—Scans of thin layer chromatograms were made using a Panax radiochromatogram scanner fitted with a propane-hydrogen gas detector head; identification of [$^{14}$C]-metabolites was made by

* Present address: Department of Pharmacology, School of Medicine, University of Bergen, Bergen, Norway.

† Present address: Bacterial Metabolism Research Laboratory, Central Public Health Laboratory, Colindale Avenue, London, N.W.9.

comparison of their chromatographic behaviour with that of authentic compounds.

*Liquid scintillation counting.*—Scintillation vials were counted in a Packard Tricarb scintillation counter and corrected for quenching by the internal channel ratios method (Bush, 1963).

*Counting of blood samples.*—0·1–0·2 ml blood was mixed in the counting vial with 1·5 ml soluene 100 (Packard Ltd)–isopropanol mixture (1 : 1). 0·5 ml of 30% $H_2O_2$ was added, the closed vial was gently shaken and the solution kept for 1 h at room temperature. After addition of 15 ml HCl: InstaGel (Packard Ltd) mixture (0·5 N HCl: InstaGel 12 : 88) and gentle shaking the sample was ready for counting. Counting efficiencies of 80–90% for $^{14}C$ and 60% for $^{3}H$ were obtained.

*Counting of tissue samples.*—0·4 ml of a 10% aqueous homogenate was mixed with 0·8 ml of water and 10 ml of InstaGel in a counting vial with gentle shaking. Duplicate samples were taken for each organ. Counting efficiencies of 80% for $^{14}C$ and 50% for $^{3}H$ were obtained.

*Synthesis of [$^{14}C$]-dimethylnitrosamine.*—A solution of [$^{14}C$]-dimethylamine hydrochloride (463 μg), unlabelled dimethylamine hydrochloride (463 μg) and sodium nitrite (50 mg) in 4 ml distilled water was acidified to pH 2 by the addition of 6N sulphuric acid. The solution was distilled to dryness and 5 μl of the distillate was analysed on a silica gel GF254 plate using hexane/ether/methylene dichloride 10/3/2 v/v as the developing solvent to separate dimethyl nitrosamine from unchanged amine (which remains at the origin). Dimethylnitrosamine accounted for 70% of the activity in the distillate; the unchanged amine was removed by the two successive additions of Amberlite 1R(120H) resin (British Drug Houses, Poole, Dorset). The pH of the solution of [$^{14}C$]-dimethylnitrosamine (DMN) was adjusted to 7 before use.

*Synthesis of [$^{3}H$]-nitrosopiperidine.*—[$^{3}H$]-N-nitrosopiperidine was synthesized by a modification of the method used by Mirvish (1970) for [$^{3}H$]-DMN. Ten ml of piperidine was redistilled and the fraction with boiling point 105–107°C was collected; 0·8 ml of this was exposed to tritium gas by the Wilzbach procedure (Wilzbach, 1957) at the Radiochemical Centre, Amersham. [$^{3}H$]-piperidine (0·1 ml) together with 0·9 ml of re-distilled piperidine was added to 100 ml of 0·1 N hydrochloric acid and distilled to remove labile tritium until only 20 ml remained in the flask. The volume was made up to 100 ml with 0·1 N hydrochloric acid and the solution again distilled until only 20 ml remained and this process was repeated until less than 10% of the counts were found in the distillate.

Sodium nitrite (6 g) was then added, the pH adjusted to 2 by the addition of 6 N sulphuric acid, and the solution distilled almost to dryness. The percentage conversion to N-nitrosopiperidine (NNP) was determined by TLC on the distillate to be 90%; the identity of the [$^{3}H$]-NNP was confirmed by mass spectrometry in comparison with the unlabelled authentic sample (Eastman Chemicals, New York).

*Experimental bladder infections in rats.*—Sprague–Dawley rats were anaesthetized with Nembutal (Abbott Laboratories), the bladder exposed and 3 stitches inserted using W328 silk sutures; 0·1 ml of a diluted broth culture of *Escherichia coli* EB 555 containing $10^7$ organisms was then injected into the bladder. After 5 days the urine was checked for the presence of infecting organisms by plating dilutions on MacConkey agar; to date all animals treated have been infected successfully. In order to confirm that the strain causing the infection was the strain introduced into the bladder (and not some opportunist strain) the organisms isolated were serotyped by the Salmonella Reference Laboratory, Colindale, and shown to be 0117.K?, the same serotype as EB 555. With time, this initial infecting strain was often superseded by a subsequent infecting organism, but this did not normally occur for at least 2 months, during which time the bladder infection was maintained at a level of at least $10^6$ organisms per ml.

*Absorption of N-nitrosamines from the rat and hamster bladder.*—Male Sprague–Dawley rats (200–250 g) or hamsters were anaesthetized by i.p. injection of thiopentone (Pentothal, Abbott) at a dose level of 90 mg/kg body weight for rats and 45 mg/kg for hamsters. A Y-shaped incision was made in the abdomen and the ureters ligated to prevent reflux to the kidney from the bladder. A catheter was placed in the left carotid artery and filled with heparinized saline (100 i.u./ml) for the collection of blood samples. A neutralized solution of nitrosamine (0·2–0·4 ml) was introduced into the bladder by means of a bladder catheter and the catheter was then clamped. 0·1 ml blood samples were removed by means of the carotid canula at $\frac{1}{2}$, 1, $1\frac{1}{2}$, 2 and $3\frac{1}{2}$ h after introduction of the nitrosamine. Heparinized saline (0·1 ml) was returned to the blood system after taking each sample.

After 4 h the animals were sacrificed, the organs removed and weighed, washed free of blood and homogenized in 10 volumes of water/isopropanol (7/3 v/v) using a Citenco homogenizer. The blood samples and tissue samples were assayed for radioactivity as described below. The tissues and organs removed were the liver, kidneys, lungs, stomach, spleen, heart, small intestine, oesophagus and bladder.

Uninfected control rats and infected rats were used for the experiments; infected rats were those in which a bladder infection had been established for 1 month. Only uninfected hamsters were used and, due to their small size, no attempt was made to insert a carotid canula; consequently, no blood samples were taken except at the end of the experiment.

*Assay of urinary nitrate.*—Urinary nitrate was assayed by the method of Kamm, McKeown and Morison Smith (1965) except that the reduction to nitrite was carried out by shaking with spongy cadmium (Elliott and Porter, 1971) instead of the slower column method.

*Assay of secondary amines.*—The secondary amines piperidine and pyrrolidine were determined in urine by the method of Asatoor and Kerr (1961). The amines were converted to their dinitrophenyl derivatives, separated by paper chromatography and eluted, then assayed spectrophotometrically.

*Assay of nitrosamines in urine.*—The urine was made to 10% with NaCl and 4% with $K_2CO_3$ then extracted 3 times with 10 ml amounts of dichloromethane; the extracts were pooled, dried over anhydrous $K_2CO_3$ and reduced in volume to 1 ml in a Kuderna-Danish evaporator. A sample of 0·1 ml was removed and assayed for nitrosamine by the method of Eisenbrand and Preussmann (1970). To positive samples 0·4 ml of hexane was added, and the azeotropic mixture evaporated to 0·1 ml for analysis by gas chromatography-mass spectrometry (GC-MS) by the method of Telling, Bryce and Althorpe (1971).

*Maintenance of animals.*—Sprague–Dawley rats were fed a diet of Oxoid 41B pellets (Oxoid) *ad libitum*. For the studies on the production of nitrosamines in the urinary bladder they were kept in Metabowl metabolism cages (Jencons, Hemel Hempstead) and given drinking water containing 5 mg/ml sodium nitrate throughout the experiment. Urine was collected in flasks covered with aluminium foil (to prevent photolytic decomposition of any nitrosamine present) and containing 0·1 ml of an 0·1% solution of merthiolate to prevent bacterial growth.

### RESULTS

### *Formation of N-nitrosamines in the rat urinary bladder*

The rats, in metabolism cages, were given water containing 5 mg/ml sodium nitrate to drink *ad libitum*; approximately 90% of the consumed dose was recovered in the urine, in agreement with our previous findings (Hawksworth and Hill, 1971*a*). After they had been consuming nitrate for 4 days, 500 $\mu$g of piperidine hydrochloride in 1 ml of distilled water (adjusted to pH 7) was administered by gastric intubation to 6 rats with bladder infections on 2 successive days and urine was collected for 24 h after each dose. Urine was also collected from 2 control, uninfected rats treated as above and from 2 infected rats given distilled water to drink instead of the nitrate solution.

When tested by the Eisenbrand–Preussmann method urine from 3/6 test animals and 0/4 controls contained nitrosamine and these results were confirmed by GC-MS; the positive urines contained 0·2 $\mu$g of NNP representing a nitrosation of 0·04% of the amine dose. When tested *in vitro* using the same dose of amine in 10 ml of broth culture and a nitrate concentration similar to that found in urine, a 0·4% conversion was obtained after 18 h incubation. Similar results were obtained when pyrrolidine was used instead of piperidine.

*Absorption of N-nitrosamines from the rat and hamster urinary bladder*

After introduction of [$^{14}$C-DMN into the rat bladder analysis of the blood samples for $^{14}$C showed that it was rapidly absorbed into the circulating blood (Fig. 1), the maximum concentration being reached after 30–60 min. There was no difference in either the rate or the extent of absorption from the bladder between rats with, and rats without, a bladder infection. When [$^{3}$H]-NNP was used the maximum level in the blood was not reached until 2–3 h after administration (Fig. 2). In the hamster experiments no carotid canulation was attempted and we have no information on the rate of absorption.

When the rats were sacrificed after 4 h 8% of the total dose of [$^{14}$C]-DMN was present in the blood, a further 8% in the major organs and 33% remained in the urine. Presumably a major part of the balance was lost as $^{14}CO_2$, since it has been shown by Swann and Magee (1968) that nitrosamine administered i.p. underwent complete metabolism to $CO_2$ in 5 h. Only 1% of the administered dose of [$^{3}$H]-NNP was present in the blood after 4 h; a further 4% was present in the major organs.

Fig. 1.—Levels of $^{14}$C in the blood of rats after the introduction of [$^{14}$C]-DMN into their urinary bladder.

Fig. 2.—Levels of $^{3}$H in the blood of rats after the introduction of [$^{3}$H]-NNP into their urinary bladder.

The distribution of the radioactivity amongst the various organs varied to some extent with the nitrosamine and the test animal. With [$^{14}$C]-DMN in the rat bladder the major organs from which radioactivity was recovered were the liver, stomach and kidney (Fig. 3); with [$^{3}$H]-NNP in the rat, the highest levels were found in the liver and kidney with very little present in any other organ (Fig. 4). After the introduction of [$^{3}$H]-N-nitrosopiperidine into the bladder of hamsters, however, a different distribution of radioactivity was seen, the levels

Fig. 3.—Distribution of $^{14}$C amongst the organs of rats 4 h after the introduction of [$^{14}$C]-DMN into their urinary bladder.

FIG. 4.—Distribution of $^{3}H$ amongst the organs of rats 4 h after the introduction of [$^{3}H$]-NNP into their urinary bladder.

of $^{3}H$ in the liver and kidney being equal, with the next highest levels being found in the stomach and small intestine and a significant amount in the lungs (Fig. 5).

In these experiments the amount of nitrosamine introduced into the bladder ranged from 2·5 to 5 $\mu$g, which is approximately the amount which would be expected to be formed in human urine, extrapolating from *in vitro* studies. Hence, the amount of absorption found may give some indication of the extent to which the absorption of nitrosamines would occur from the human bladder.

FIG. 5.—Distribution of $^{3}H$ amongst the organs of hamsters 4 h after the introduction of [$^{3}H$]-NNP into their urinary bladder.

DISCUSSION

We have now demonstrated the production of N-nitrosamines from the secondary amines piperidine and pyrrolidine *in vivo* in the infected rat urinary bladder; both of these amines are present in normal human urine. However, very high amine concentrations were used in order to obtain enough nitrosamine to allow GC-MS confirmatory analysis; we could not then go on to use a labelled amine followed by TLC scanning as a means of detecting nitrosamine formed from small amounts of amine because there is a normal urinary metabolite formed from both these cyclic amines with the same TLC behaviour as the N-nitrosamine. Such an interfering metabolite was not produced from dimethylamine and a single experiment using low amine concentrations indicated that the yield of nitrosamine was higher, in terms of the percentage of amine nitrosated, at the low concentration. Experiments using low nitrate concentrations were not possible because of the very variable nitrate content of the Oxoid 41B rat pellets. It will be necessary to find a nitrate-free food source and this is under investigation.

Obviously the production of nitrosamines in the bladder is of interest only if it can also be demonstrated that such products are not wholly excreted in the urine but may be absorbed into the circulating blood. We have demonstrated that after the introduction of DMN into the bladder 16% of the dose may be detected in the form of $^{14}C$ in the blood and tissues only 4 h later. It must be remembered that DMN is rapidly metabolized in the rat to $CO_2$ so that the 16% detected represents only a fraction of that which had been absorbed. The distribution of label amongst the various organs was similar to that reported by others following administration of N-nitrosamines i.p., and this would be expected since, in both cases, the nitrosamine was first absorbed into the blood then transported to the organs by essentially the same route.

These results indicate that nitrosamines may be produced *in vivo* and that such nitrosamines may be absorbed and thus contribute to human cancer. Nitrosamines have been reported in the urine of humans with urinary tract infections (Brooks *et al.*, 1972). There is as yet no clear evidence that nitrosamines, whether of endogenous or exogenous origin, are of any relevance to human cancer and there are no data on the target organ of any nitrosamine in the human. We have suggested that there is some evidence that the target organ of DMN is the human stomach (Hill, Hawksworth and Tattersall, 1973) and the necessary clues should be obtainable, from epidemiological studies, to the target organ of other nitrosamines.

We are grateful to Dr Marion Hicks of Middlesex Hospital Medical School for showing us the bladder catheter technique. This work was financially supported by the Cancer Research Campaign and by the British Nutrition Foundation.

### REFERENCES

Asatoor, A. M. & Kerr, D. N. S. (1961) Amines in Blood and Urine in Relation to Liver Disease. *Clin. chim. Acta*, **6**, 149.

Brooks, J. B., Cherry, W. B., Thacker, L. & Alley, C. C. (1972) Analysis by Gas Chromatography of Amines and Nitrosamines produced *in vivo* and *in vitro* by *Proteus mirabilis*. *J. infect. Dis.*, **126**, 143.

Bush, E. T. (1963) General Applicability of the Channels Ratio Method of Measuring Liquid Scintillation Counting Efficiencies. *Analyt. Chem.*, **35**, 1024.

Eisenbrand, G. & Preussmann, R. (1970) Eine neue Methode zur kolorimetrischen Bestimmung von Nitrosaminen nach Spaltung der N-nitrosogruppe mit Bromwasserstoff in Eisessig. *Arzneimittel-Forsch.*, **20**, 1513.

Elliott, R. J. & Porter, A. G. (1971) A Rapid Cadmium Reduction Method for the Determination of Nitrate in Bacon and Curing Brines. *Analyst*, **96**, 522.

Friedman, M. A., Greene, E. J. & Epstein, S. S. (1972) Rapid Gastric Acid Absorption of Sodium Nitrite in Mice. *J. pharm. Sci.*, **61**, 1492.

Hawksworth, G. M. & Hill, M. J. (1971*a*) The Formation of Nitrosamines by Human Intestinal Bacteria. *Biochem. J.*, **122**, 28P.

Hawksworth, G. M. & Hill, M. J. (1971*b*) Bacteria and the N-nitrosation of Secondary Amines. *Br. J. Cancer*, **25**, 520.

Hill, M. J. & Hawksworth, G. M. (1972) Bacterial Production of Nitrosamines *in vitro* and *in vivo*. *N-nitroso compounds: analysis and formation.* I.A.R.C. Scientific Publication No. 3, p. 116.

Hill, M. J., Hawksworth, G. M. & Tattersall, G. (1973) Bacteria, Nitrosamines and Cancer of the Stomach. *Br. J. Cancer*, **28**, 562.

Kamm, L., McKeown, G. G. & Morison Smith, D. (1965) New Colorimetric Method for the Determination of Nitrate and Nitrite in Baby Foods. *J. A.O.A.C.*, **48**, 892.

Mirvish, S. S. (1970) Kinetics of Dimethylamine Nitrosation in Relation to Nitrosamine Carcinogenesis. *J. natn. Cancer Inst.*, **44**, 633.

Sander, J. (1968) Nitrosaminsynthese durch Bakterian. *Z. physiol. Chem.*, **349**, 429.

Sander, J., Schweinsberg, F. & Menz, H. P. (1968) Untersuchungen uber die Entstehung cancerogener Nitrosamine im Magen. *Z. physiol. Chem.*, **349**, 1691.

Sen, N. P., Smith, D. E. & Schwinghamer, L. (1969) Formation of N-nitrosamines from Secondary Amines and Nitrite in Human and Animal Gastric Juice. *Food, & Cosmet. Toxicol.*, **7**, 301.

Swann, P. F. & Magee, P. N. (1968) Nitrosamine induced Carcinogenesis. The Alkylation of Nucleic Acids of the Rat by N-methyl-N-nitrosourea, Dimethylnitrosamine, Dimethylsulphate and Methylmethane Sulphonate. *Biochem., J.*, **110**, 39.

Telling, G. M., Bryce, T. A. & Althorpe, J. (1971) Use of Vacuum Distillation and Gas Chromatography-Mass Spectrometry for Determining Low Levels of Volatile Nitrosamines in Meat Products. *J. agric Fd Chem.*, **19**, 937.

Wilzbach, K. E. (1957) Tritium Labelling by Exposure of Organic Compounds to Tritium Gas. *J. Am. Chem. Soc.*, **79**, 1013.